# An Overview of Nd:YAG Laser Capsulotomy 

**Published:** 2014

**Authors:** Eyyup Karahan, Duygu Er, Suleyman Kaynak

**Affiliations:** 1 Egepol Hospital, Department of Ophthalmology, Izmir, Turkey; 2 Dokuz Eylul University, Department of Ophthalmology, Izmir, Turkey

**Keywords:** Nd:YAG Laser, Capsulotomy, Posterior capsule opacification (PCO)

## Abstract

It has been revealed that posterior capsule opacification (PCO) is the most common delayed complication of cataract surgery. On the other hand, Nd:YAG laser capsulotomy is accepted as standard treatment for PCO. Although, Nd:YAG laser capsulotomy is a noninvasive and safe treatment it carries risk of some complications. Using less total energy and performing smaller capsulotomies are effective choices to decrease complications after Nd:YAG capsulotomy. The purpose of this review is to look through the complications associated with Nd:YAG laser capsulotomy, and the effect of capsulotomy size and used total energy on such complications.

## INTRODUCTION

Posterior capsule opacification (PCO) is the most common delayed complication of cataract surgery (1). The incidence of PCO was reported to be 20.7% at two years and 28.5% at 5 years after cataract surgery (2). In the early 1980s, the application of Neodymium:yttrium-aluminum-garnet (Nd:YAG) laser capsulotomy as treatment for PCO was presented by Aron-Rosa (3) and Fankhauser (4). Nd:YAG laser capsulotomy showed itself to be an effective alternative to surgical discission, avoiding such complications as endophthalmitis and vitreous loss (5,6).

Improvement in visual acuity after Nd:YAG laser capsulotomy in patients with significant PCO has been well documented (7–9). Improvements in glare and contrast sensitivity may also be important outcome measures for many patients (10–12). Although Nd:YAG laser capsulotomy is accepted as standart treatment for PCO and has been found to be safe and effective, it is not without complications, some of which can be sight-threatening such as retinal edema and detachment (13). Several studies have described damages in the intraocular lens (IOL), increased intraocular pressure (IOP), glaucoma, retinal hemorrhage, iritis, vitreous prolapse, corneal injury, vitritis, pupil blockage, hyphema, cystoid macular edema, retinal detachment (RD), IOL dislocation or exacerbation of endophthalmitis (3,6,14–16).

Some recent studies have been concentrated to the influence of capsulotomy size and total energy level on complications after Nd:YAG laser capsulotomy (17–19).

The purpose of this review is to look through the complications associated with Nd:YAG laser capsulotomy, and the effect of capsulotomy size and used total energy on such complications.

## COMPLICATIONS ASSOCIATED WITH ND:YAG LASER CAPSULOTOMY

*IOL Movement and Refractive Changes*

There are several reports of displaced IOLs after laser treatment (21–24). Levy et al. reported two instances of hydrogel implant dislocation into vitreous following Nd:YAG laser capsulotomy (21). Using dual-beam partial coherence interferometry the procedure of Nd:YAG laser capsulotomy has been shown to induce a small but measurable backward movement of the IOL. They stated that the larger capsulotomy openings induce greater backward movement, and recommend small openings to avoid this complication. No siginificant refractive change was reported in this study (25). However, Thornval and Naeser (26) failed to observe this effect. In our recently reported study we found that the hyperopic shift was higher in patients with capsulotomy size larger than 3.9 mm when compared with patients with smaller capsulotomy sizes. The hyperopic shift was progressive until 4 weeks in larger capsulotomy group. We recommended to prescript new spectacles at least 1 week or 4 weeks if the capsulotomy is large after Nd:YAG laser capsulotomy (17). Zaidi et al., were also recorded a significant hyperopic shift which was especially important 1 week after Nd:YAG laser capsulotomy (27). Also the magnitude of shift can be affected by IOL style. The hyperopic shift was found higher with plate haptic implants than with polymethyl methacrylate and three piece foldable lenses (25).

*IOL Damage/Pitting*

Hassan KS et al. has noted IOL pitting 19.8% in a study of 86 eyes (28) and Haris WS noted 11.7% significant marks on IOL during laser capsulotomy in 342 eyes (29). Khanzada et al. (30) reported the range of 9.4% (30 eyes) in 320 eyes. The retro-focusing of laser aiming beam can reduce the risk of IOL damage.

*Iritis/Uveitis*

Keates et al. found iritis persisting in 0.4% and vitritis persisting in 0.7% after a 6-month postoperative period (13). Chambless, in a study with an average follow-up period of 7 months, found persistent anterior uveitis in 1.4% of the patients (6). Gore et al. reported that 33.5% of patients had iritis after Nd:YAG laser capsulotomy manifested as cells and flare in the anterior chamber on slit lamp examination. They were given topical steroid, and reaction had subsided leaving no delayed complication (31). In summary, transient anterior chamber flare may be seen post-laser treatment; persistent iritis or vitritis is rare.

*Rise of Intraocular Pressure*

The most common complication of posterior capsulotomy is increased IOP. Different explanations which have been given for the pressure rise following Nd: YAG laser treatment include the deposition of debris in the trabecular meshwork, (32,33) pupillary block, (34,35) and inflammatory swelling of the ciliary body or iris root associated with angle closure (36). Despite the prophylactic treatment, increased IOP was reported in 15% to 30% of patients in several studies (37,38). Keates et al. (13) found elevation of IOP in 0, 6% of his patients, whereas Stark et al. (8) reported that the elevation of IOP was 1.0% after Nd:YAG capsulotomy. Ge et al. (39) found that the rise in IOP was more pronounced in patients with glaucoma in those who experienced a higher rise of IOP within hour of capsulotomy. However, Shani et al. (40) could not find any elevation of IOP and postulated that healthy pseudophakic eyes do not show elevation of IOP after Nd: YAG laser capsulotomy. Ficker et al. (31) noted 13 patients to have IOP over 23 mmHg and 9 patients to have IOP between 30–48 mmHg, within 2–3 hours after laser capsulotomy. In this group of 24 patients there was a tendency for IOP to rise when higher pulse energies were used, particularly when these exceeded 1.5 mg, and the raised IOP was controlled with antiglaucoma therapy. Ari et al. (20) underlined that the severity and duration of increased IOP and macular thickness were less when a total energy level less than 80 mg is used.

In our study, one patient (2.7%) in small size group and three patients (9.3%) in larger capsulotomy size group had mild elevation of IOP one week after Nd: YAG laser capsulotomy. Rise in IOP was higher than previous studies. Previous studies did not give any information about the capsulotomy size. So, a comparison of rises in IOP with previous studies is not appropriate. More capsule particles released with larger capsulotomies might be the reason of higher rates of elevation in larger capsulotomy group (17).

*Cystoid Macular Edema*

Cystoid macular edema (CME) occurs after intraocular surgical procedures, trauma, and a variety of other inflammatory conditions affecting the retina. The etiology of CME following Nd: YAG laser capsulotomy most likely involves movement of the vitreous cavity and vitreous damage, which results in the release of inflammatory mediators. Vitreoretinal traction caused by the procedure may also play a part (41).

Previous studies have investigated changes of macular thickness after Nd: YAG laser capsulotomy. Although some of the previous studies have reported CME, many of them found no significant changes in macular thickness following Nd: YAG laser capsulotomy (42–47).

Lewis et al. found a low rate of CME when capsulotomy was delayed for over 6 months from the initial IOL implant date (48). Ari et al. (18) evaluated how different energy levels of Nd: YAG laser capsulotomy affect macular thickness. They divided patients into two groups based on the energy levels used in Nd: YAG laser capsulotomy. They found that both groups had increased macular thickness compared to preoperative levels; macular thickness measurements of the patients treated with high energy levels were significantly greater compared to low energy levels. In another study a series of 897 Nd: YAG laser capsulotomies were reviewed for the complications of CME. After Nd: YAG laser capsulotomy, 11 patients developed CME. The numbers of laser pulses and energy delivered were not risk factors (19).

In our study, energy levels were similar in both small size and large size capsulotomy groups. Comparison of two groups with respect to macular thickness did not reveal any difference preoperatively or 1 week, 4 weeks or 12 weeks postoperatively. There was a significant thickening in macular thickness at 1 week in both groups; this difference was not statistically significant between groups. The mean macular thicknesses were decreased to preoperative levels at 4 and 12 weeks measurements (17).

*Retinal Tear and Detachment*

The risk for RD after Nd: YAG laser capsulotomy is estimated to be 4-fold that of the risk after uneventful surgery without a capsulotomy (49,50). Raza (51) reported 11 patients (2%) of RD after Nd: YAG laser capsulotomy. Steinert et al. (19) reported that eight patients of 897 patients treated with Nd: YAG laser capsulotomy developed RD.

Retrospective analysis of data based on Medicare claims in the US suggests that Nd:YAG laser capsulotomy is associated with a significantly elevated risk for RD, stronger associations were found for a history of RD or lattice degeneration, an axial length greater than 24.0 mm, and posterior capsule rupture during surgery (49). Several other retrospective studies confirm the higher risk of RD after capsulotomy in eyes with intraoperative complications, axial myopia, and vitreoretinal pathology (52–55); however, 2 studies show no association in the absence of these risk factors (56,57).

The precise mechanisms that lead to retinal breaks and RD after Nd:YAG laser capsulotomy are not known, Sheard et al. designed a study to determine whether RD after Nd:YAG laser capsulotomy is due to a greater incidence of posterior vitreous detachment (PVD) than in controls and whether vitreous status at the time of capsulotomy is useful in predicting the risk for RD. The prevalence of PVD was significantly higher in eyes after extra-capsular cataract extraction and IOL implantation than in Phakic eyes independent of Nd: YAG laser capsulotomy. Capsulotomy was not associated with a significantly greater incidence of new PVD, they concluded that the presence or absence of PVD at the time of capsulotomy is not helpful in assessing the risk for RD in the first year after laser treatment (58).

*Other Complications*

Pupillary block glaucoma (8) as well as aqueous misdirection syndrome, (59) macular hole, (6) retinal hemorrhage, (8) spreading of endocapsular low-grade endophthalmitis, (60) and secondary closure of capsulotomy aperture (61) are other complications that have been reported in isolation.

## DISCUSSION/CONCLUSION

Nd: YAG laser capsulotomy is fast and noninvasive procedure with immediate improvement. Although, it is noninvasive and considered safer than surgical approach it carries the risk of some complications. Some recent studies including our study observed the effects of capsulotomy size and laser energy levels on postcasulotomy complications (17–19).

Capsulotomy size is important as patients subjected to lower amounts of laser energy for perhaps a smaller capsulotomy may benefit from fewer complications of RD, IOP rise, (62,63) and perhaps to less CME (64). Risk of IOL dislocation may be significantly less, especially with plate haptic silicone IOLs. In our study, despite energy levels were similar in small and large capsulotomy groups hyperopic shift, IOP rise and increased macular thickness was found to be lesser in patients with a smaller capsulotomy size.

Another parameter that is believed to be important is laser energy level. Ari et al. reported that IOP rise and rise in macular thickness were higher with higher energy levels. However, Steiner et al. reported that the numbers of laser pulses and energy delivered, not risk factors for the development of cystoid macular edema.

In conclusion, some complications especially rise in IOP and macular thickness seems to be unavoidable after Nd: YAG laser capsulotomy. Using less total energy and performing smaller capsulotomies are practical choices to decrease complications after Nd: YAG capsulotomy.
